# Chromosome 9p21.3 polymorphism in a Chinese Han population is associated with angiographic coronary plaque progression in non-diabetic but not in type 2 diabetic patients

**DOI:** 10.1186/1475-2840-9-33

**Published:** 2010-08-06

**Authors:** Wei Wang, Wenhui Peng, Xianling Zhang, Lin Lu, Ruiyan Zhang, Qi Zhang, Lingjie Wang, Qiujing Chen, Weifeng Shen

**Affiliations:** 1 Department of Cardiology, Rui Jin Hospital, Shanghai Jiaotong University School of Medicine, Shanghai 200025, People's Republic of China; 2 Institute of Cardiovascular Diseases, Shanghai Jiaotong University School of Medicine, Shanghai 200025, People's Republic of China

## Abstract

**Background:**

We sought to explore the association of variant rs1333049 on chromosome 9p21.3 with coronary artery disease (CAD) and angiographic plaque progression in non-diabetic and type 2 diabetic patients.

**Methods:**

Genotyping and quantitative coronary angiography (QCA) were performed in 2046 Chinese Han patients (1012 diabetic cases) undergoing coronary angiography; 430 of them received repeat angiographic studies at 1-year follow-up.

**Results:**

CC genotype at rs1333049 on chromosome 9p21.3 was associated with CAD (unadjusted OR 1.524, *p *= 0.001 and adjusted OR 1.859, *p *= 0.005, respectively). However, CC genotype had no magnified association with CAD in diabetic patients (OR 1.275, *p *= 0.150) compared with non-diabetic counterparts (OR 1.446, *p *= 0.020) after adjusting for conventional risk factors. During angiographic follow-up, non-diabetic patients (n = 280) had significant decrease in minimal lumen diameter and increase in percent diameter stenosis among the three genotypes (*p *= 0.005 and *p *= 0.038, respectively), demonstrating that CC or GC genotype carriers had a more severe plaque progression than GG genotype carriers. In patients with type 2 diabetes (n = 150), although plaque progression was more severe than that in non-diabetic counterparts, no relations existed between plaque progression and genotypes. Rs1333049 was an independent determinant of plaque progression for non-diabetic (OR 3.468, *p *= 0.004 and OR 4.339, *p *= 0.002 for GC and CC genotype, respectively) but not for diabetic patients (OR 0.529, *p *= 0.077 and 0R 0.878, *p *= 0.644 for GC and CC genotype, respectively).

**Conclusions:**

This study demonstrates a significant association of homozygous CC genotype of rs1333049 on chromosome 9p21.3 with CAD in Chinese Han population. Rs1333049 polymorphism is an independent determinant for coronary plaque progression in non-diabetic but not in type 2 diabetic patients.

## Background

Recent genome-wide scanning has implicated chromosome 9p21.3 as a novel locus conferring susceptibility to coronary artery disease (CAD), myocardial infarction and cardiac death [1-5], which is independent of traditional risk factors including gender, age, obesity, smoking, hypertension and hyperlipidemia [6,7]. Several studies showed that chromosome 9p21.3 was a vital genetic region with different independent loci of SNPs related to either diabetes or CAD [8-11]. The risk of CAD associated with 9p21 variant was increased in the presence of poor glycemic control in type 2 diabetes [8]. Also, as one of the risk equivalents of CAD, patients with diabetes often had an increased atherosclerotic burden and inflammatory process in the coronary artery tree [12-14]. Thus, we hypothesized that certain locus on chromosome 9p21.3 might have its effect in a common pathway of diabetes and CAD. In a recent study, Chen et al revealed no association between coronary atherosclerotic plaque progression and polymorphism on chromosome 9p21.3 in Caucasian population [15]. Admittedly, this cross-section study was not designed to seek possible association of 9p21.3 with CAD in a special diabetic population. Furthermore, genetic effect of variant rs1333049 on chromosome 9p21.3 on angiographic coronary disease progression in Chinese patients remains unclear. Therefore, the present case-control study was conducted to examine whether this locus influences angiographic plaque progression in Chinese Han non-diabetic and type 2 diabetic patients.

## Methods

### Patients

The study protocol was approved by the hospital Ethics Committee, and written informed consents were obtained from all subjects.

The study population consisted of 2046 Chinese Han patients (1012 diabetic and 1034 non-diabetic cases) undergoing coronary angiography between March 2004 and December 2007 for the evaluation of suspected or established CAD (luminal diameter narrowing ≥50%); 430 of them received repeat angiography at 1-year follow-up. Type 2 diabetes was defined as a fasting plasma glucose level ≥7.0 mmol/L or non-fasting plasma glucose level≥11.1 mmol/L, or taking oral hypoglycemic drugs or receiving parenteral insulin therapy. Patients with type 1 diabetes were excluded by measuring C peptide, and excluded were also those with chronic viral or bacterial infection, tumor, or immune system disorders. Diagnosis of hypertension was based on the presence of elevated systolic (≥140 mmHg) and/or diastolic (≥90 mmHg) blood pressure, or current use of antihypertensive medications for one year before admission. Patients were diagnosed as hyperlipidemia if serum levels of total cholesterol (TC)>5.7 mmol/l (220 mg/dl), triglycerides (TG) >1.7 mmol/l (150 mg/dl), low-density lipoprotein cholesterol (LDL-C) >3.64 mmol/L (140 mg/dl), or high-density lipoprotein cholesterol (HDL-C) <0.91 mmol/L (35 mg/dl).

### Coronary angiography and quantitative analysis

Coronary angiography was performed using standard Judkins techniques or through radial approach, and all major coronary arteries were carefully imaged on at least two orthogonal views. Quantitative coronary analysis (QCA) was performed (TERRA, GE, USA) by two experienced interventional cardiologists, who were unaware of clinical information of the patients. For those with coronary angiography at baseline and 1-year follow-up, all coronary arteries intervened with percutaneous coronary intervention (PCI) were excluded to avoid inclusion of post-PCI neointimal hyperplasia or restenosis [16]. The coronary artery segments analyzed included all those plaques with a reference diameter ≥ 1.5 mm and a stenosis ≥ 20% at baseline and those with new lesions at follow-up in non-PCI intervened artery. Using the outer diameter of the contrast-filled catheter as the calibration, the minimal lumen diameter (MLD) in diastole was measured from multiple projections. Atherosclerotic plaque progression was diagnosed if one of the following criteria was met: (1) ≥10% diameter reduction of a pre-existing stenosis ≥50%; (2) ≥30% diameter reduction of a stenosis <50%; (3) progression of any stenosis to total occlusion, or (4) development of a new stenosis ≥30% in a previously normal segment [17]. A new coronary lesion was defined as a stenosis that was not apparent on the initial angiogram or was <20% in diameter stenosis but that narrowed by≥0.4 mm in MLD at the follow-up angiogram [18]. Coronary artery score was calculated from per-patient average of the MLD of all the measured segments in observed coronary artery, and cumulative coronary obstruction was the sum of all percent diameter stenosis in standard index unit (50% = 0.50) [19]. Change of QCA measurements was defined as baseline QCA measurement minus follow-up measurement.

### Biochemical investigation

Blood samples were collected after an overnight fasting in all patients and were stored at -80°C. Serum glucose, hemoglobin A_1c _(HbA_1c_), insulin levels, blood urea nitrogen (BUN), creatinine, and lipid profiles (TC,LDL-C,HDL-C,TG) were measured (HITACHI 912 Analyzer, Roche Diagnostics, Germany). Serum high sensitivity C-reactive protein (hsCRP) level was determined using a high-sensitivity ELISA kit (Biocheck Laboratories, Toledo, OH, USA) with a linear range of 0.62-119.3 mg/L and an inter-assay CV <7.5%.

### Genotyping

Genomic DNA was extracted from peripheral blood leucocytes by standard phenol-chloroform extraction. Genotyping was performed with TaqMan SNP allelic discrimination by means of an ABI 7900HT (Applied Biosystems, Foster City, CA, USA), in 384-well format. The TaqMan Assay kit was purchased from Applied Biosystems (Foster City, CA, USA). Genotypes were determined with the same method as previously described [20], in which primers of rs1333049 polymorphism were TCACTACCCTACTGTCATTCCTCAT and TTGCTTACCTCTGCGAGTGG, and probes were VIC-CAACAGTTCAAAAGCA and FAM-AACAGTTGAAAAGCA. Data were analyzed using the ABI Prism SDS software version 2.1.

### Statistical analysis

Continuous variables are presented as mean ± standard deviation, and categorical data are summarized as frequencies or percentages. Normal distribution of continuous variable was evaluated with Kolmolgorov-Smirnov test. For categorical variables, differences between groups were evaluated by the chi-square test. Differences among genotypes were analyzed by one-way analysis of variance (ANOVA) followed by post-hoc analysis (Bonferroni's correction) for comparison between groups. Odds ratios (ORs) of CAD were first estimated by chi-square test and then adjusted by traditional risk factors for CAD including gender, age, hypertension, hyperlipidemia, smoking status and diabetes (all factors with *p *< 0.05 between CAD and non-CAD in the study population). ORs of covariates determining plaque progression were estimated using a multivariable logistic regression model. A 2-sided probability level of ≤0.05 was considered significant. All analyses were done with SPSS for Windows 13.0 (SPSS Inc, Chicago, Illinois, USA).

## Results

### Baseline characteristics of the study population

As expected, patients with CAD were older and more male gender, and had more risk factors for CAD including smoking, hypertension, hyperlipidemia and diabetes and higher serum hsCRP level than those without CAD (all *p *< 0.05; data not shown). Despite similar medical treatments, patients with plaque progression had more diabetes, elevated serum levels of 2 h plasma glucose and hsCRP, and reduced HDL-C than those without plaque progression (Table 1).

**Table 1:** Baseline clinical characteristics and biochemical assessments of plaque progression study

	CAD		
	
	Plaque Progression (n = 137)	No Plaque Progression (n = 293)	*P*
Men/Female (n)	97/40	208/85	0.864
Age (years)	64.13 ± 7.80	65.51 ± 10.13	0.299
Cigarette smoking (%)	61(44.5%)	139(47.4%)	0.604
Hypertension (%)	65 (47.4%)	130 (44.4%)	0.512
Hyperlipidemia (%)	36 (26.3%)	65 (22.1%)	0.466
Diabetes (%)	69 (43.0%)	81 (28.0%)	0.033
BMI (kg/m^2^)	24.63 ± 3.46	24.05 ± 3.24	0.189
Fasting plasma glucose (mmol/L)	5.49 ± 1.88	5.99 ± 2.00	0.255
2 h plasma glucose (mmol/L)	13.40 ± 4.20	11.29 ± 3.66	0.039
HbA_1c _(%)	6.79 ± 1.48	6.64 ± 1.43	0.315
hsCRP (mg/ml)	19.35 ± 13.63	11.87 ± 9.22	<0.001
Total cholesterol (mmol/L)	4.77 ± 1.04	4.84 ± 1.16	0.631
Triglyceride (mmol/L)	1.93 ± 1.53	2.02 ± 1.32	0.319
LDL-C (mmol/L)	2.80 ± 0.79	2.84 ± 0.85	0.672
HDL-C (mmol/L)	1.09 ± 0.31	1.27 ± 0.27	0.015
apoA (g/L)	1.22 ± 0.20	1.27 ± 0.22	0.156
apoB (g/L)	0.97 ± 0.22	0.95 ± 0.23	0.674
Lipoprotein (a) (g/L)	0.25 ± 0.18	0.23 ± 0.16	0.422
BUN (mmol/L)	5.49 ± 1.89	5.88 ± 1.65	0.078
creatinine (mg/L)	91.51 ± 23.16	89.78 ± 20.08	0.534
Statin (%)	126 (92.1%)	262 (89.5%)	0.475
ACEI or ARB (%)	114 (83.3%)	231 (79.0%)	0.285
β-blocker (%)	59 (43.1%)	121 (50.9%)	0.448
Antiplatelet (%)	123 (90.1%)	215 (90.3%)	0.656
Insulin (%)	36 (26.2%)	30 (12.7%)	0.069
Oral anti-diabetic drugs (%)	49 (35.7%)	111 (46.5%)	0.144

Data are number (%) and mean ± SD;

Abbreviations: CAD, coronary artery disease; BMI, body mass index; HbA1c, glycosylated hemoglobin; hsCRP, high sensitivity C-reactive protein; HDL-C, high-density lipoprotein cholesterol; LDL-C, low-density lipoprotein cholesterol; BUN, blood urea nitrogen.

### Association between rs1333049 polymorphism and CAD

The genotype frequencies of rs1333049 were in Hardy-Weinberg equilibrium in patients with and without CAD (all *p *> 0.05, data not shown). CC genotype of rs1333049 was associated with CAD in overall patients with unadjusted OR 1.524, 95% CI 1.192-1.949, *p *= 0.001 and adjusted OR 1.859, 95% CI 1.212-2.852, *p *= 0.005, respectively (Table 2). Further analysis showed that CC genotype was not significantly associated with CAD in diabetic patients (OR 1.275, 95% CI 0.843-1.930, *p *= 0.150) compared with non-diabetic counterparts (OR 1.446 95% CI 1.145-1.826, *p *= 0.020).

**Table 2:** Multivariable analysis of independent determinants for coronary artery disease in the whole population

Variables	Unadjusted OR (95% CI)	*p*	Adjusted OR (95% CI)	*p*
Rs1333049 (GC vs. GG)	1.199 (0.966-1.487)	0.099	1.105 (0.755-1.619)	0.607
Rs1333049 (CC vs. GG)	1.524 (1.192-1.949)	0.001	1.859 (1.212-2.852)	0.005
Gender (male vs. female)	2.534 (2.132-3.011)	<0.001	2.516 (2.107-3.224)	<0.001
Age (years)	2.093 (1.760-2.489)	<0.001	1.952 (1.627-2.341)	<0.001
Hypertension (yes vs. no)	1.589 (1.347-1.875)	<0.001	1.490 (1.251-1.774)	<0.001
Hyperlipidemia (yes vs. no)	1.187 (0.952-1.473)	0.119	1.274 (1.008-1.608)	0.042
Smoking (yes vs. no)	2.242 (1.893-2.655)	<0.001	1.837 (1.527-2.211)	<0.001
Diabetes (yes vs. no)	1.873 (1.571-2.233)	<0.001	1.780 (1.470-2.157)	<0.001

Risk factors which are adjusted include gender, age, hypertension, hyperlipidemia, smoking status and diabetes.

### Association between rs1333049 polymorphism and plaque progression

Biochemical measurements and angiographic features with respect to various genotypes are listed in Table 3. White blood cell (WBC) and neutrophile counts were significantly higher in CC genotype carriers. There were no significant differences in changes of MLD, percent diameter stenosis and number of new coronary lesions among the three genotypes in the whole population at follow-up angiography (all *p *> 0.05) (Table 3). In non-diabetic patients, risk C allele of rs1333049 was related to degree of MLD reduction (0.25 ± 0.49 mm for CC genotype, 0.20 ± 0.35 mm for GC genotype, and 0.05 ± 0.35 mm for GG genotype, respectively, *p *= 0.005) (Table 4). Similar findings were observed for change of percent diameter stenosis, coronary artery score and cumulative coronary obstruction (*p *= 0.038, 0.004 and 0.025, respectively). Although diabetic patients had more severe plaque progression than non-diabetic counterparts, no relations existed between plaque progression and genotypes (all *p *> 0.05) (Table 4).

**Table 3:** Changes in biochemical and angiographic measurements during follow-up

	Rs1333049			
	GG (n = 122)	GC (n = 200)	CC (n = 108)	*p*
**Change of Biochemical measurements**				

Total cholesterol (mmol/L)	0.64 ± 1.17	0.41 ± 1.12	0.53 ± 1.14	0.228
LDL-C (mmol/L)	0.27 ± 0.98	0.24 ± 0.90	0.34 ± 0.86	0.623
HDL-C (mmol/L)	-0.05 ± 0.49	-0.01 ± 0.22	0.01 ± 0.19 ^a^	0.422
Triglyceride (mmol/L)	0.41 ± 1.92	0.10 ± 0.86 ^a^	0.16 ± 1.06 ^b^	0.106
apoA (g/L)	0.10 ± 0.15	0.09 ± 0.17	0.08 ± 0.16	0.029
apoB (g/L)	0.41 ± 0.37	0.39 ± 0.30	0.40 ± 0.34	0.639
Lipoprotein (a) (g/L)	-0.01 ± 0.08	-0.02 ± 0.09	-0.01 ± 0.12	0.873
Fast glucose (mmol/L)	0.39 ± 1.22	0.42 ± 2.48	0.66 ± 1.58	0.487
HbA_1c _(%)	0.52 ± 1.54	0.40 ± 1.32	0.27 ± 1.44	0.419
White blood cells (× 10 ^9^/L)	0.75 ± 2.02	0.92 ± 1.94 ^a^	1.46 ± 1.87 ^a^	0.012
Neutrophile (× 10 ^9^/L)	0.51 ± 1.64	0.66 ± 1.98	1.13 ± 1.80 ^a^	0.025
Lymphocyte (× 10 ^9^/L)	0.21 ± 0.66	0.21 ± 0.64	0.29 ± 0.54	0.463
Smoking status				
Non-smoker (%)	67 (54.9%)	109 (54.5%)	54 (49.2%)	0.309
Smoker with cessation (%)	30 (24.6%)	49 (24.5%)	30 (27.7%)	0.474
Smoker without cessation (%)	25 (20.5%)	42 (21.0%)	24 (23.1%)	0.532
Angiographic features				
New coronary lesion	0.09 ± 0.29	0.07 ± 0.26	0.11 ± 0.32	0.385
Change of MLD (mm)	0.18 ± 0.46	0.26 ± 0.44	0.29 ± 0.50	0.151
Change of diameter stenosis (%)	-5.14 ± 15.87	-8.82 ± 16.03	-9.38 ± 14.89	0.081
Coronary artery score (mm)	0.05 ± 0.03	0.16 ± 0.07	0.21 ± 0.11	0.188
Cumulative coronary obstruction	-0.03 ± 0.09	-0.20 ± 0.13	-0.31 ± 0.21	0.076

MLD, minimal lumen diameter; other abbreviations are listed in table 1

a < 0.05 compared with GG

b <0.01 compared with GG

**Table 4:** Plaque progression in different genotypes of rs1333049

Non-DM (N = 280)	GG (n = 90)	GC (n = 114)	CC (n = 76)	*p*
New coronary lesion	0.05 ± 0.22	0.07 ± 0.27	0.11 ± 0.32	0.347
Change of MLD (mm)	0.05 ± 0.35	0.20 ± 0.35 ^a^	0.25 ± 0.49 ^b^	0.005
Change of diameter stenosis (%)	-3.44 ± 13.80	-7.38 ± 13.70	-8.93 ± 14.64 ^a^	0.038
Coronary artery score (mm)	0.01 ± 0.02	0.12 ± 0.04 ^a^	0.16 ± 0.07 ^b^	0.004
Cumulative coronary obstruction	-0.02 ± 0.11	-0.17 ± 0.09	-0.28 ± 0.13 ^a^	0.025

**DM (N = 150)**	**GG (n = 32)**	**GC (n = 86)**	**CC (n = 32)**	
				
New coronary lesion	0.19 ± 0.40	0.07 ± 0.23	0.13 ± 0.34	0.173
Change of MLD (mm)	0.34 ± 0.53	0.39 ± 0.53	0.46 ± 0.56	0.513
Change of diameter stenosis (%)	-9.20 ± 19.61	-10.86 ± 18.77	-10.67 ± 15.74	0.908
Coronary artery score (mm)	0.14 ± 0.07	0.22 ± 0.11	0.29 ± 0.15	0.152
Cumulative coronary obstruction	-0.05 ± 0.10	-0.25 ± 0.18	-0.36 ± 0.27	0.770

MLD, minimal lumen diameter.

a < 0.05 compared with GG

b <0.01 compared with GG

### Determinants of plaque progression

Multivariable logistic regression analysis revealed that gender (male), hypertension, genotypes carrying allele C, low HDL-C and high hsCRP were independently associated with plaque progression in non-diabetic patients during 1-year follow-up (OR 3.468 95% CI 1.504-8.000, *p *= 0.004 for GC genotype and OR 4.339 95% CI 1.740-10.821, *p *= 0.002 for CC genotype). Meanwhile, gender (male), age, hyperlipidemia, smoking and high hsCRP, but not risk genotype of rs1333049, were independent determinants of plaque progression in diabetic patients (Table 5).

**Table 5:** Multivariable logistic regression analysis of independent determinants for plaque progression

	HR	95% CI	*p*		HR	95% CI	*p*
**Non-diabetes**				**Diabetes**			

rs1333049 (GC vs. GG)	3.468	1.504-8.000	0.004		0.529	0.685-1.474	0.077
rs1333049 (CC vs. GG)	4.339	1.740-10.821	0.002		0.878	0.278-2.814	0.644
Gender (male vs. female)	2.912	1.184-7.161	0.020		1.807	1.615-3.100	0.007
Age (y)	0.993	0.914-1.025	0.051		0.932	0.876-0.995	0.027
Hypertension	2.129	1.066-4.251	0.032		2.287	0.840-6.223	0.105
Hyperlipidemia	2.110	0.732-4.148	0.324		6.057	1.922-22.145	0.003
Smoking	1.301	0.638-2.656	0.469		3.165	1.008-9.938	0.048
Triglyceride	0.880	0.601-1.298	0.886		1.130	0.580-2.119	0.720
Total cholesterol	0.531	0.247-1.429	0.528		0.431	0.254-1.116	0.077
LDL-C	1.422	0.497-2.104	0.701		1.749	0.556-2.599	0.378
HDL-C	0.471	0.106-0.877	0.030		0.214	0.005-0.915	0.037
Creatinine	1.104	0.975-1.156	0.226		1.009	0.989-1.029	0.361
Baseline hsCRP	1.302	1.115-1.522	<0.001		1.283	1.211-1.309	<0.001
Use of statins	1.401	0.644-3.492	0.562		1.709	0.590-4.952	0.317
BMI	1.028	0.925-1.143	0.608		1.025	0.889-1.181	0.734

Abbreviations are as in Table 1.

## Discussion

This study demonstrates that SNP of rs1333049 on chromosome 9p21.3 increases the susceptibility to CAD in Chinese Han population and confers a magnified risk of coronary plaque progression in non-diabetic patients.

Several genome-wide association studies have shown that different genetic variations on chromosome 9p21.3 were associated with increased risk of diabetes, CAD and sudden cardiac death in the general population [2,8-10]. As one of the risk equivalents of CAD, diabetes increased atherosclerotic burden and inflammatory process in the coronary artery tree [12-14]. Doria et al reported an interaction between poor glycemic control and 9p21 locus on risk of CAD in type 2 diabetes [8]. However, we observed that homozygous CC genotype of rs1333049 was not strongly associated with CAD in diabetic population in this study, which was in accordance with the findings from a prospective meta-analysis that no interaction existed between diabetes and rs1333049 polymorphism in CAD development [7]. Similarly, there was a magnified risk of C allele for coronary plaque progression in non-diabetic but not in diabetic patients, implying that polymorphism on chromosome 9p21 might have effects on CAD development in a novel biological pathway other than interaction with diabetes or glucose metabolism.

Genetic variation on chromosome 9p21 has been reported to be associated with progression of carotid but not coronary atherosclerosis in Caucasian population [15,21]. Besides, specific polymorphisms in the chromosome 9p21.3 region that were shown to be associated with CAD in genome-wide analyses might not be related to clinical and angiographic outcomes after implantation of drug-eluting stents in the coronary arteries [22]. Thus, this study was the first to show an association of SNP on chromosome 9p21.3 with CAD and angiographic plaque progression in a special population. In addition, variant at chromosome 9p21 was associated with recurrent myocardial infarction and cardiac death after acute coronary syndrome, serving as a predictive factor of perioperative myocardial injury after coronary artery bypass graft surgery [23,24]. Taken together, this genetic factor might partially increase the risk of CAD by promoting atherosclerosis development or plaque instability.

The high risk of haplotype at 9p21.3 was shown to overlap with exons 13 to 19 of ANRIL [25-28], which was expressed in atheromatous plaques and served as a functional enhancer of cellular proliferation and inflammation [25,27]. And, variants on 9p21.3 had a role in regulation of cardiac cyclin dependent kinase inhibitor 2A/2B (CDKN2A/2B) expression by altering the dynamics of vascular cell proliferation [29]. As white blood cells especially neutrophiles, an important cell line in vascular inflammation, were significantly increased in CC genotype carriers in this study, the relationship between 9p21.3 and inflammation might be a possible explanation for angiographic plaque progression. Further functional analyses are needed to clarify possible pathways in which this SNP on chromosome 9p21.3 contributes to development of CAD and plaque progression.

Although diabetic patients had more severe plaque progression than non-diabetic counterparts, risk genotype of rs1333049 was not an independent determinant of plaque progression in diabetes. The difference in association between variant rs1333049 on chromosome 9p21.3 and plaque progression in diabetic and non-diabetic patients suggested that CAD in diabetes might be mediated through a pathway other than polymorphism on 9p21.3, and perhaps the role of other risk factors exceeded that of variants on 9p21.3 in conferring more severe plaque burden for diabetic patients.

This study had several limitations. First, all patients received medical treatments during follow-up, which could affect the natural course of the disease and plaque progression. However, since patients with or without plaque progression received similar medical treatment which was listed in Table 1 and no significant change of medical treatment was made before the result of the follow-up angiography, these could help to decrease the influence of treatment to certain extent. Second, case-control studies had advantages for identifying disease related genes, but they were limited to detect gene-environment interactions [30]. A prospective cohort study is needed to better illustrate the role of genetic and environmental factors as well as their interactions in CAD development.

## Conclusions

Polymorphism on chromosome 9p21.3 is significantly associated with CAD in Chinese Han population, and also contributes to coronary plaque progression in non-diabetic patients. Thus, SNP scanning on 9p21.3 should been done to select the patients with potential risk of CAD or angiographic plaque progression in non-diabetic population. Early medical intervention or close follow-up of non-diabetic patients with risk genotype is of equal importance to primary or secondary prevention for CAD in diabetes.

## Competing interests

The authors declare that they have no competing interests.

## Authors' contributions

WW, WP, XZ, LW and QC carried out the molecular genetic studies and DNA extraction, participated in the sequence alignment, genotyping study and drafted the manuscript. WW, LL, RZ and QZ participated in the design of the study and performed the statistical analysis. WS conceived of the study, and participated in its design and coordination. All authors read and approved the final manuscript.
